# Ultra-high-*Q* resonances in plasmonic metasurfaces

**DOI:** 10.1038/s41467-021-21196-2

**Published:** 2021-02-12

**Authors:** M. Saad Bin-Alam, Orad Reshef, Yaryna Mamchur, M. Zahirul Alam, Graham Carlow, Jeremy Upham, Brian T. Sullivan, Jean-Michel Ménard, Mikko J. Huttunen, Robert W. Boyd, Ksenia Dolgaleva

**Affiliations:** 1 School of Electrical Engineering and Computer Science, University of Ottawa, Ottawa, ON Canada; 2 Department of Physics, University of Ottawa, Ottawa, ON Canada; 3 National Technical University of Ukraine “Igor Sikorsky Kyiv Polytechnic Institute”, Kyiv, Ukraine; 4 Iridian Spectral Technologies Inc., Ottawa, ON Canada; 5 Photonics Laboratory, Physics Unit, Tampere University, Tampere, Finland; 6 Institute of Optics and Department of Physics and Astronomy, University of Rochester, Rochester, NY USA

**Keywords:** Nanophotonics and plasmonics, Optics and photonics, Nanocavities

## Abstract

Plasmonic nanostructures hold promise for the realization of ultra-thin sub-wavelength devices, reducing power operating thresholds and enabling nonlinear optical functionality in metasurfaces. However, this promise is substantially undercut by absorption introduced by resistive losses, causing the metasurface community to turn away from plasmonics in favour of alternative material platforms (e.g., dielectrics) that provide weaker field enhancement, but more tolerable losses. Here, we report a plasmonic metasurface with a quality-factor (*Q*-factor) of 2340 in the telecommunication C band by exploiting surface lattice resonances (SLRs), exceeding the record by an order of magnitude. Additionally, we show that SLRs retain many of the same benefits as localized plasmonic resonances, such as field enhancement and strong confinement of light along the metal surface. Our results demonstrate that SLRs provide an exciting and unexplored method to tailor incident light fields, and could pave the way to flexible wavelength-scale devices for any optical resonating application.

## Abstract (web summary)

Metallic nanostructures are useful in many optical devices due to their nonlinear properties and responses to interaction with light. Here the authors demonstrate a metasurface of gold nanoparticle arrays with ultra-narrow surface lattice resonances of high quality-factor that operates in the telecommunication band.

## Introduction

Metallic nanostructures are essential to many applications in photonics, including biosensing^1^, spectroscopy^2,3^, nanolasing^4^, all-optical switching^5^, nonlinear optical processes^6^, and metasurface technologies^7–9^. These plasmonic elements form flexible components with geometry-dependent responses and have many desirable properties, such as the possibility to confine light to sub-wavelength scales and large local-field enhancements^9,10^. Metals also possess intrinsic nonlinear optical constants that are many orders of magnitude larger than dielectric materials^11^.

When structured at the sub-wavelength scale^8,9,12^, individual nanostructures exhibit localized surface plasmon resonances (LSPRs), where electromagnetic fields couple to the free-electron plasma of a conductor at a metal–dielectric interface^6,10^. Depending on its shape, an individual nanoparticle may be polarized by an incident light beam, acting as a lossy dipole antenna^13^ and trapping light for a short period of time. In contrast to other photonic resonant devices such as whispering gallery mode resonators, microring resonators, or photonic crystals^14–16^, resonating dipoles in a metasurface can easily be accessed by a beam propagating in free space and require only a sub-wavelength propagation region for operation. Therefore, a plasmonic metasurface resonator enables a series of specialized optical responses, including phase-matching-free nonlinear optical effects^6,17^, strongly localized field enhancements^9^, multi-mode operation^18^, and a spatially localized optical response^7^. Such a metasurface with a large quality factor (*Q*-factor) could be used as a cavity for applications that need increased light–matter interactions, small mode volumes, large field enhancements, and large optical nonlinearities, such as an ultra-flat nano-laser with a large transverse mode size^4,19^ or frequency conversion applications (e.g., nonlinear harmonic generation^20^ or THz-wave generation^21^). One frequently cited limitation of LSPR-based metasurfaces are their low *Q*-factors (e.g., *Q* < 10) due to the intrinsic Ohmic losses present in metals at optical frequencies^10,22–24^. As the *Q*-factor is related to the light–matter interaction time as well as to enhancements to the electric field, it is typically desirable to maximize this quantity^14^. Low *Q*-factors therefore make many potential applications of plasmonics-based metasurface devices impractical, and new methods for obtaining large *Q*-factor resonances in a metasurface have long been sought after.

The optical response of coupled plasmonic nanoresonators has been a topic of intense study^25^. Notably, plasmonic metasurfaces of large periodically arranged nanostructures support collective resonances called surface lattice resonances (SLRs)^26–32^. Here the individual responses from the surface plasmons of many individual nanostructures form a collective response that couples to in-plane diffraction orders of the periodic array^26,30^. As a consequence, a relatively high-*Q* resonance can emerge at an optical wavelength *λ*_SLR_ ≈ *nP*, close to the product of the refractive index of the background medium *n* and the lattice period *P*^26,32^. Recent theoretical studies of this platform have predicted *Q*-factors on the order of 10^3^ by properly engineering the dimensions of the individual nanostructures and the period of the lattice^31–33^, hinting at the possibility of combining the aforementioned benefits of metals with long interaction times provided by high *Q*-factors. However, to date, the highest experimentally observed *Q*-factor in an SLR-based metasurface is 430^34^. The disparity between theory and experiment has been attributed to a variety of reasons, including poor spatial coherence of light beams^28,35^, small array sizes^30,31,36^, fabrication imperfections^30,31^, and the addition of an adhesion layer^37^.

Inspired by this discrepancy, here we perform a detailed investigation to determine the dominant factors that most drastically affect the observed *Q* of an SLR-based metasurface: the nanostructure geometry, the array size, and the spatial coherence of the probing light source. Using the results of this study, we demonstrate a plasmonic metasurface capable of supporting ultra-high-*Q* SLRs.

## Results

**Fig. 1:**
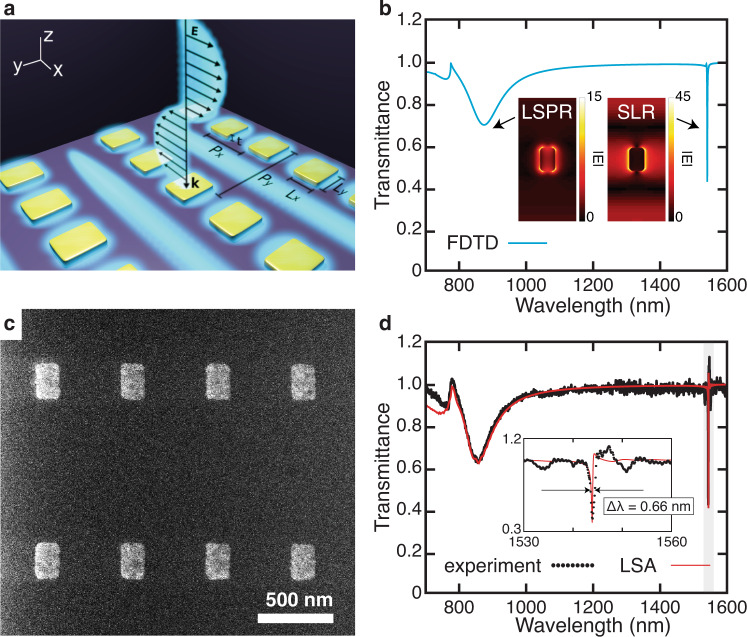
High-*Q* metasurface nanocavities using arrays of plasmonic nanostructures. **a** Schematic of the metasurface consisting of a rectangular array of rectangular gold nanostructures. Here *L*_*x*_ = 130 nm, *L*_*y*_ = 200 nm, *t* = 20 nm, *P*_*x*_ = 500 nm, and *P*_*y*_ = 1060 nm. The blue shaded regions illustrate the electric field, reproducing the mode structure in the inset of **b**. **b** Numerical (FDTD) calculations of the transmission spectrum of this metasurface for *x*-polarized light. Both the LSPR and the SLR are observed in these results. Inset: The simulated magnitude of the electric field ∣*E*∣ for the entire unit cell of both LSPR and SLR modes in the *x*–*y* plane that bisects the nanoparticles. The color bar indicates the relative magnitude when normalized to the incident plane wave. **c** Helium ion microscopic image of the fabricated metasurface prior to cladding deposition. **d** Measured transmission spectrum (black dots) and fits to semi-analytic calculations (LSA, red line). Inset: Zoomed plot of the highlighted region in **d**. Fitting the measurement to a Lorentzian function yields a linewidth of Δ*λ* = 0.66 nm, corresponding to *Q* = 2340 (see Supplementary Sec. S1: *Q*-factor extraction).

The metasurface in consideration consists of a rectangular array of rectangular gold nanostructures embedded in a homogeneous silica glass (*n* ~ 1.45) environment (Fig. 1a). The lattice constant *P*_*y*_ = 1060 nm was selected to place the SLR wavelength in the telecommunication window; *P*_*x*_ = 500 nm was reduced from a square lattice, increasing the nanoparticle density and consequently increasing the extinction ratio of the resonance. The overcladding is carefully matched to the substrate material to ensure a symmetric cladding index, as it has been shown that the *Q* of an SLR may be affected by the homogeneity of the environment^26,38,39^. As shown by the numerical predictions in Fig. 1b, for an *x*-polarized beam, this metasurface is expected to support an LSPR at *λ*_LSPR_ = 830 nm and an SLR of the first type around *λ*_SLR_ = 1550 nm (See Supplementary Sec. S2: SLR type). The SLR linewidth is substantially narrower than that of the LSPR, corresponding to a much higher *Q*-factor. Incidentally, the inset field profiles in Fig. 1b also reveal that the SLR provides a more significant field enhancement, with $| {E}_{\max }({\lambda }_{{\rm{SLR}}})| \sim 3| {E}_{\max }({\lambda }_{{\rm{LSPR}}})|$. Figure 1c shows an image of the fabricated device with dimensions matching those of the simulations. The measured transmission spectra are presented in Fig. 1d, closely matching the predicted spectrum. Notably, the full width at half-maximum of the linewidth is only Δ*λ* = 0.66 nm, corresponding to a *Q*-factor of *Q* = 2340. This value exceeds the record for plasmonic metasurfaces by an order of magnitude^34,37,40^ and is among the highest reported in a metasurface. It is roughly within a factor of two of semi-analytic calculations performed using the lattice sum approach (LSA), where *Q* ~ 5000 (see “Methods” for details). In order to observe this value for the *Q*-factor, both the metasurface and the measurement apparatus needed to be arranged with a few considerations in mind, which we describe in greater detail below.

### The role of nanoparticle polarizability

First, the individual structures need to be engineered to exhibit the appropriate response at *λ*_SLR_. The optical response of a nanostructure can be approximated using the polarizability of a Lorentzian dipole,

1 $$\alpha (\omega )=\frac{{A}_{0}}{\omega -{\omega }_{0}+i\gamma },$$

where *A*_0_ is the oscillator strength, *ω*_0_ = 2*πc*/*λ*_LSPR_ corresponds to the nanoparticle resonance frequency, and *γ* is the damping term. These quantities all depend on the particle geometry^12^ (here the length *L*_*y*_ and width *L*_*x*_ of a rectangular bar). The contribution of the particle lattice to the polarizability can be introduced using the LSA^39,41^:

2 $${\alpha }^{* }(\omega )=\frac{\alpha (\omega )}{1-{\epsilon }_{0}\alpha (\omega )S(\omega )},$$

where α^*^(*ω*) is known as the effective polarizability of the entire metasurface and *S*(*ω*) corresponds to the lattice sum. This latter term depends only on the arrangement of the lattice. An SLR appears approximately where *S*(*ω*) exhibits a pole, at *ω*_SLR_ = (2*πc*/*λ*_SLR_). At this spectral location, the individual responses of all of the nanostructures contribute cooperatively^41^.

Equation (2) may be used to predict the optical response of the entire metasurface, including the behavior of its many resonances, as a function of the geometry of its nanostructures (see “Methods”): by changing the geometry of a nanostructure^12,42^, its individual resonance wavelength *λ*_LSPR_, oscillator strength *A*_0_, and damping constant *γ* are all modified. In turn, adjusting these values changes the polarizability of the nanostructures throughout the spectrum, including at the SLR wavelength *α*(*ω*_SLR_), and therefore also the response of the entire metasurface at this wavelength α^*^(*ω*_SLR_). Here we adjust the above parameters by changing the dimensions of the nanostructures (see “Methods”), while the parameters could be alternatively modified by considering altogether different nanostructure shapes, such as nanorings, nanorods, or core–shell nanoparticles^42^. By contrast, the spectral location of the SLR wavelength is dictated mainly by the lattice period and the background index *λ*_SLR_ ≈ *nP*^32,43,44^. In other words, the lattice configuration governs the presence of the SLR, and the nanostructure geometry dictates its coupling efficiency to free space. Indeed, recent theoretical studies in this platform have shown *Q*-factors on the order of 10^3^ by properly selecting the dimensions of the individual nanostructures^31,33^.

**Fig. 2:**
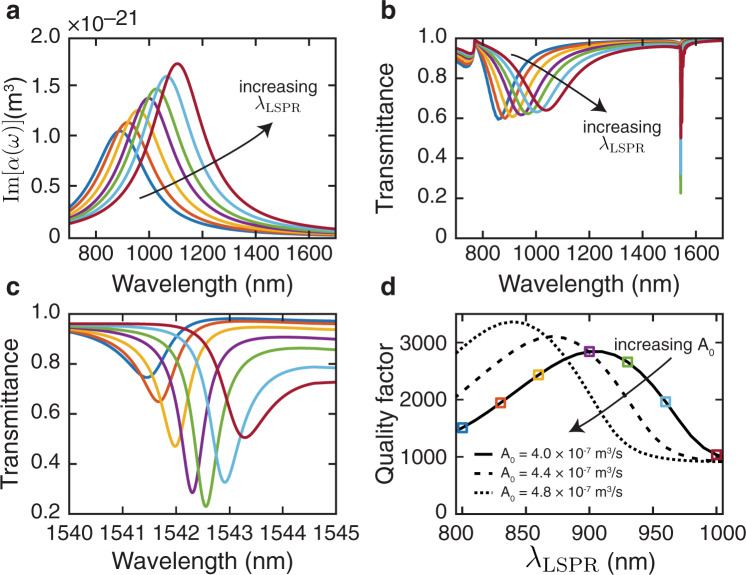
Coupling to a surface lattice resonance. The colors in **a**–**d** are consistent, corresponding to the same type of nanoparticle. **a** The imaginary part of the individual particle polarizability for various nanostructures with increasing resonance wavelength *λ*_LSPR_, holding both the oscillator strength *A*_0_ and the damping term *γ* fixed. Here *λ*_LSPR_ is tuned from 800 to 1000 nm. **b** Simulated broadband transmission spectra for gold nanostructure arrays as a function of tuning *λ*_LSPR_. By tuning the LSPR wavelength, the extinction factor of the SLR is observed to change near *λ* = 1542 nm. While *λ*_LSPR_ changes dramatically, the SLR wavelength *λ*_SLR_ does not change much. **c** Zoomed-in plot of the SLR in **b**. **d** The *Q*-factor of the surface lattice resonance as a function of *λ*_LSPR_ for various oscillator strengths *A*_0_. The optimal LSPR wavelength for a high-*Q* SLR changes as a function of *A*_0_. The oscillator strength *A*_0_ increases from 3.98 × 10^−7^ to 4.77 × 10^−7^ m^3^/s, roughly corresponding to a 20% increase in the particle volume. The squares indicate the *Q* values extracted from the curves in **c**.

We reproduce this dependence in this platform explicitly by plotting the calculated transmission of a metasurface (see “Methods”) as a function of nanostructure resonance wavelength *λ*_LSPR_ (Fig. 2). (The dependence of the SLR behavior on particle dimensions, which is connected to the resonance wavelength, is also demonstrated using full-wave simulations in Supplementary Sec. S3: Dependence of SLR behavior on particle dimensions.) Here we hold the oscillator strength *A*_0_ and damping term *γ* constant and slowly increase the nanoparticle resonance wavelength *λ*_LSPR_. Note that the resonance position differs slightly from the position of the dip due to the incorporation of a long-wavelength correction^45^. In Fig. 2b, c, the SLR wavelength does not change substantially from its location around *λ*_SLR_ = 1542 nm; however, the extinction ratio Δ*T* and the linewidth Δ*λ* of the resonance change dramatically. In Fig. 2d, we plot the extracted *Q*-factors for these SLRs and for other values of *A*_0_, as well (see Supplementary Sec. S1: Q-factor extraction for the fits). Based on well-established relationships between nanoparticle geometry and polarizability^10,46^, this *A*_0_ range corresponds to a change in nanoparticle volume of roughly 20%. We find that, for every given value of *A*_0_, there is a corresponding *λ*_LSPR_ for which light couples optimally to the lattice resonance at *λ*_SLR_ and produces the highest *Q*-factor. The optimal conditions are therefore found in the balance between increasing *α* relative to *P*_*y*_ (i.e., increasing coupling strength) and maintaining a large spectral gap between *λ*_LSPR_ and *λ*_SLR_ (i.e., limiting Ohmic losses associated with metallic nanoparticles). The trade-off between coupling and loss is a traditional one for optical resonators and is reproduced in the SLR-based metasurface platform^47^.

### Effect of array size

Next, we study the dependence of the *Q*-factor on the array size. For certain metasurfaces, it has already been predicted that larger array sizes lead to better device performance^36,48^. This dependence makes some intuitive sense—since high-*Q* operation requires low absorption losses, we are required to operate the device far from the LSPR. However, at a sufficiently far operating wavelength, the scattering cross-section is also small, resulting in each antenna scattering very weakly. Consequently, far from the LSPR, one requires a sufficiently large number of scatterers to build up the resonance. Equivalently, the standing wave mode in an SLR consists of counter-propagating surface waves; therefore, a larger array provides an expanded propagation length in the cavity to support these modes.

**Fig. 3:**
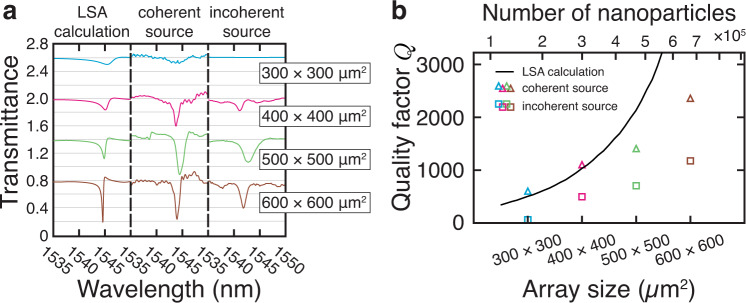
Effects of array size and spatial coherence of light source. **a** Calculated and measured (using coherent and incoherent sources) transmission spectra for identical metasurface arrays of varying size (from top to bottom: 300 × 300, 400 × 400, 500 × 500, and 600 × 600 μm^2^, respectively). The spectra are offset for clarity, and each vertical division corresponds to an increment of Δ*T* = 0.2 in transmittance. **b** The *Q*-factors extracted from Lorentzian fits to the calculations and to the measurements shown in **a**. An increase in the number of nanostructures in the array results in an increase in the estimated *Q*-factors. Additionally, the observed *Q*-factor is globally larger for each array when measured using the coherent source.

To examine the dependence of *Q* on the number of nanostructures explicitly, we fabricated and characterized a series of devices of increasing array size. Figure 3 shows the resulting transmission spectra, as well as their corresponding semi-analytic predictions. The observed *Q*-factors increase monotonically as a function of array size (Fig. 3b—see Supplementary Sec. S1: *Q*-factor extraction for the fits). In the smallest array (300 × 300 μm^2^), the SLR is almost imperceptible. This trend might help explain the relatively low *Q* values observed in previous studies^9,30,31,36^ where array sizes were typically no larger than 250 × 250 μm^2^, likely due to the relatively slow write-speed of the electron-beam lithography process necessary for fabrication^26,37^. By contrast, our devices have array sizes reaching up to 600 × 600 μm^2^ (see Supplementary Sec. S4: Image of the device).

### The role of spatial coherence

Finally, it is of critical importance to consider all aspects of the characterization system in order to get an accurate measurement of the *Q*-factor. In particular, we have found that the spatial coherence of the probe beam was critical to obtaining a clean measurement of the dip in transmission indicating a resonance. A spatially coherent beam, such as a laser, excites every region of the metasurface in phase, producing a resonance feature that is both deeper and narrower compared to using a spatially incoherent source. Additionally, the higher-order modes of the lattice are more sensitive to angular variance in the measurements, leading to broader peaks when using incoherent sources^35^. Furthermore, in our particular experiment, the transmitted signal from our coherent supercontinuum source was both brighter and could also be better collimated than our incoherent thermal source. Therefore, the light collected from the metasurface array could be isolated with a smaller pinhole in the image plane, selecting the signal coming from nanostructures at the center of the array with a more uniform collective response.

In Fig. 3, we compare the performance of the metasurface when illuminated using different light sources: a broadband supercontinuum laser (i.e., a well-collimated coherent source), and a tungsten-halogen lamp. The comparison between these measurements indicates that the *Q* increases with the coherence of the light source—using the thermal light source reduces the *Q*-factor by a factor of 2–5 when compared to the laser. Additionally, it decreases the resonance coupling strength, as is evident from the reduced extinction ratio of the SLRs. Figure 3b summarizes the *Q*-factors extracted from these measurements and compares them to numerical predictions. LSA calculations predict that *Q*-factors increase as a function of array size; this trend continues for both smaller and larger devices than those probed experimentally. Note that, even when using an incoherent source, the largest array still produces a very large *Q*-factor (*Q* ~ 1000). The observation of such a high *Q* using an incoherent source reinforces the validity of our aforementioned metasurface design criteria—that is, the importance of the choice of nanostructure geometry and of the array size.

In some of the measurements, the value for the normalized transmittance can be seen to exceed unity (i.e., *T* > 1). We speculate that this is because the nanostructures aid in coupling to the substrate, reducing the reflections from the first interface.

## Discussion

Despite promising results, Fig. 3b also highlights some discrepancies between the simulation and the experiment for the largest arrays, notably reducing the measured *Q*-factors. This disparity could be due to multiple reasons, which we enumerate below. First, the prediction produced by the LSA might be overestimating the *Q* by assuming that each nanoparticle is excited with a constant-valued local field. This assumption cannot be entirely correct for a Gaussian beam and a finite array, where particles closer to the boundaries of the array feel a weaker local field than the particles near the center. Second, the fabrication procedure produces stitching errors, which become more important for larger arrays. This added disorder might contribute to the reduction in *Q*. Lastly, the *Q*-factors might be limited due to additional measurement considerations, such as the finite coherence length of the light source or imperfections with the collimation.

In this work, we only looked into rectangular nanoparticles in rectangular lattices. Based on LSA calculations and the discrete-dipole approximation (DDA) used in previous work^26,39,44,49,50^, it is evident that any particle geometry (e.g., cylindrical, rectangular, or triangular) that can be approximated by dipoles with the same Lorentzian parameters *A*_0_, *λ*_LSPR_, and *γ* will yield an identical SLR *Q*-factor. For nanoparticles that cannot be modeled by dipoles—regardless of the particle geometry—the SLR *Q*-factor will be the same provided that the polarizability at *λ*_SLR_ remains the same. Regarding different lattice configurations, the spectral responses of other lattice geometries such as hexagonal, orthorhombic, and kagome are likely to be different than the rectangular lattice design we have adopted. However, lattice sums can be computed for these regular lattices, and therefore they can also be treated using our method. Therefore, strategies presented in this work are largely blind to the specific lattice arrangements, and its conclusions will be helpful in obtaining resonances with large-*Q* factors in other geometries.

The *Q*-factors for the type of device presented here could be further increased, however, by considering larger arrays or by further optimizing the nanostructure dimensions—instead of rectangles, a more intricate nanostructure shape could tailor the Lorentzian dipole coefficients *A*_0_, *λ*_LSPR_, and *γ* more independently to allow for optimal coupling and higher extinction ratios. These shapes include L-shaped antennas^51^, split-ring resonators^52^, and others that also exhibit higher-order moments^53,54^. Alternatively, a nanoparticle with a large aspect ratio could increase coupling to more neighboring particles using out-of-plane oscillations^44^. Finally, the metasurface shown here can be combined with other established methods to enable multiple simultaneous resonances^39,50,55^.

Table 1 contains a short survey of the literature on metasurface nanocavities. Other than the reported *Q*-factors, we have included, when available, information that is relevant to compare their work against ours, such as the operating wavelength, the material platform, the array size, and the type of light source used. Our work demonstrates the highest *Q* by an order of magnitude among metasurfaces with plasmonic components and is exceeded only by metasurfaces that incorporate a bound state in the continuum (BIC).

**Table 1:** Summary of experimentally obtained *Q*-factors in metasurfaces.

Mechanism	*Q*	*λ* (nm)	Material	Light source	Array size (μm^2^)	Reference
**SLR**	**2340**	**1550**	**Au NPs**	**Supercontinuum**	**600** **×** **600**	**This work**
LSPR	<10	700	Au NPs	Tungs.-halogen lamp	3000 × 3000	^61^
SLR	25	930	Au NPs	Collimated source	135 × 135	^27^
SLR	30	850	Au NPs	Tungs.-halogen lamp	3000 × 3000	^61^
SLR	40–60	600	Au NPs	Ellipsometer	200 × 200	^28^
SLR	60	800	Au NPs	Tungs.-halogen lamp	35 × 35	^26^
SLR	150	764	Au NPs	Tungs.-halogen lamp	N/A	^62^
SLR	230	900	Au NPs	Tungs.-halogen lamp	~10,000 × 10,000	^63^
SLR	300	1500	Au nanostripes	Tungs.-halogen lamp	300 × 100	^40^
SLR	330	648	Ag NPs	Tungs.-halogen lamp	2500 × 2500	^37^
SLR	430	860	Au NPs	Tungs.-halogen lamp	1000 × 1000	^34^
Mirror image	200	5000	ITO nanorods	Collimated source	N/A	^35^
EIT	483	1380	Si	Tungs.-halogen lamp	225 × 240	^64^
Fano resonance	65	THz	Al particles	THz laser	10,000 × 10,000	^65^
Fano resonance	100	THz	Au Assym. NPs	FTIR	150 × 150	^66^
Fano resonance	350	1000	Si	N/A	N/A	^67^
Fano resonance	600	1000	GaAs	N/A	N/A	^67^
BIC	2750	825	GaAs	Laser	60 × 108	^68^
Quasi-BIC	18,511	1588.4	Si	Laser	15 × 15	^69^

The results presented in this work are in bold.

*Q* quality factor, *λ* resonance wavelength, *NP* nanoparticle, *SLR* surface lattice resonance, *LSPR* localized surface plasmon resonance, *EIT* electromagnetically induced transparency, *BIC* bound state in the continuum.

To summarize, we have fabricated and experimentally demonstrated a plasmonic metasurface nanoresonator with a high *Q*-factor, which is in excellent agreement with numerical predictions. Our work presents the experimental demonstration of a high-*Q* plasmonic metasurface nanoresonator with an order-of-magnitude improvement over prior art (see Table 1). We have found that the observed *Q*-factor obtained from an SLR may be limited by a poor choice of nanostructure dimensions, a small array size, or poor spatial coherence of the source illumination; we hypothesize that one or many of these factors may have been the cause for the low *Q*-factors reported in previous experiments featuring SLRs. Additionally, our device follows simple design principles that can be easily expanded upon to enable multiple resonances to fully tailor the transmission spectrum of a wavelength-scale surface. Our result highlights the potential of SLR-based metasurfaces and expands the capabilities of plasmonic nanoparticles for many optical applications.

## Methods

### Simulations

#### Finite-difference time domain (FDTD)

Full-wave simulations were performed using a commercial three-dimensional FDTD solver. A single unit cell was simulated using periodic boundary conditions in the in-plane dimensions and perfectly matched layers in the out-of-plane dimension. The structures were modeled using fully dispersive optical material properties for silica^56^ and for gold^57^. Minimal artificial absorption (${\rm{Im}}(n)\ \sim \ 1{0}^{-4}$) was added to the background medium to reduce numerical divergences.

#### Lattice sum approach

The LSA is a variant of the DDA method^58^. It is a semi-analytic calculation method that has been found to produce accurate results for plasmonic arrays^39,41,44^. The main assumption in LSA when compared to DDA is that the dipole moments of all interacting nanoparticles are assumed to be identical^44^. The main benefit of using LSA for our application compared to alternatives such as FDTD is its capability to model finite-sized arrays with an arbitrary number of nanostructures, by assuming that the overall response of the array closely follows the responses of the nanoparticles at the center of the array. By comparing simulations performed using the LSA against the DDA, this assumption has also been found to be quite accurate^44^. Its rapid simulation time makes it a useful tool for iterating many simulations to study trends and behaviors of entire metasurfaces, especially for finite array effects, such as the effect of array size on the *Q*-factor.

Using the LSA approach, the dipole moment **p** of any particle in the array is written as

3 $${\bf{p}}=\frac{{\epsilon }_{0}\alpha (\omega ){{\bf{E}}}_{{\rm{inc}}}}{1-{\epsilon }_{0}\alpha (\omega ){{S}}(\omega )}\equiv {\epsilon }_{0}{\alpha }^{* }(\omega ){{\bf{E}}}_{{\rm{inc}}},$$

where the effect of inter-particle coupling is incorporated in the lattice sum ${{S}}$ and α^*^ is the effective polarizability. This equation produces Eq. (2) in the main text. The calculations presented in this work also incorporate a modified long-wavelength correction^45^:

4 $$\alpha (\omega )\to \frac{{\alpha }_{{\rm{static}}}(\omega )}{1-\frac{2}{3}i{k}^{3}{\alpha }_{{\rm{static}}}(\omega )-\frac{{k}^{2}}{l}{\alpha }_{{\rm{static}}}(\omega )},$$

where *k* is the wavenumber in the background medium *k* = (2*πn*/*λ*) and *l* is the effective particle radius. Also here, minimal artificial absorption (${\rm{Im}}(n)=6\times 1{0}^{-4}$) was added to the refractive index *n* = 1.452 of the background medium to reduce numerical divergences associated with the approach when considering large arrays^41^. We set *l* = 180 nm for all calculations. The static polarizability of the nanoparticle is given by

5 $${\alpha }_{{\rm{static}}}(\omega )=\frac{{A}_{0}}{\omega -{\omega }_{0}-i\gamma },$$

where *A*_0_ is the oscillator strength, *ω*_0_ = 2*πc*/*λ*_LSPR_ corresponds to the nanoparticle resonance frequency, and *γ* is the damping term.

For a planar array of *N* dipoles, the lattice sum term ${{S}}$ is

6 $${{S}}(\omega )=\mathop{\sum }\limits_{j=1}^{N}\frac{\exp ({\rm{i}}k{r}_{j})}{{\epsilon }_{0}{r}_{j}}\left[{k}^{2}+\frac{(1-{\rm{i}}k{r}_{j})(3{\cos }^{2}{\theta }_{j}-1)}{{r}_{j}^{2}}\right],$$

where *r*_*j*_ is the distance to the *j*th dipole and *θ*_*j*_ is the angle between **r**_*j*_ and the dipole moment **p**.

The optical transmission spectra can be obtained by using the optical theorem, ${\rm{Ext}}\propto k{\rm{Im}}({\alpha }^{* })$^59^:

7 $$T(\omega )=1-\frac{4\pi k}{{P}_{x}{P}_{y}}{\rm{Im}}[{\alpha }^{* }(\omega )],$$

where *P*_*x*_ and *P*_*y*_ are the lattice constants along the *x* and *y* dimensions, respectively.

To produce the plots in Fig. 1d, we performed an LSA calculation using the following parameters for the single dipole: *λ*_LSPR_ = 780 nm; *A*_0_ = 3.46 × 10^−7^ m^3^/s, *γ* = 8.5 × 10^13^ s^−1^. LSA parameters were determined by matching to FDTD data. The lattice constants were *P*_*x*_ = 500 nm and *P*_*y*_ = 1067.5 nm. The total array size was 600 × 600 μm^2^, corresponding to *N*_*x*_ = 1200 × *N*_*y*_ = 562 nanostructures, respectively. The LSA calculations in Fig. 3 used these same parameters but varied the total number of nanostructures.

To calculate the figures in Fig. 2a, c, we performed a series of LSA calculation using the following parameters for the particle: *A*_0_ = 3.98 × 10^−7^ m^3^/s, *γ* = 1/[2*π*(2.1 fs)] ≈ 7.6 × 10^13^ s^−1^. The dipole resonance wavelengths *λ*_LSPR_ were 800, 833, 866, 900, 933, 966, and 1000 nm, respectively. Based on the performed FDTD simulations, these resonance wavelengths could correspond to rectangular gold nanostructures with widths of *L*_*x*_ = 110, 120, 130, 140, 150, 160, and 170 nm, respectively, if *L*_*y*_ = 190 nm, and *t* = 20 nm. (Note that, in the main text, *L*_*y*_ = 200 nm). See Supplementary Sec. S3: Dependence of SLR behavior on particle dimensions for the corresponding simulations. The lattice constants were *P*_*x*_ = 500 nm and *P*_*y*_ = 1060 nm, respectively. The total array size was 600 × 600 μm^2^, corresponding to *N*_*x*_ = 1200 × *N*_*y*_ = 567 nanostructures, respectively. To obtain Fig. 2d, a series of LSA calculations were performed for many values of *λ*_LSPR_ ranging from 800 to 1000 nm, and the *Q*-factors were extracted from the results using a fit to a Lorentzian. The curves in Fig. 2d come from repeating this procedure with oscillator strengths of *A*_0_ = 3.98 × 10^−7^, 4.38 × 10^−7^, and 4.77 × 10^−7^ m^3^/s.

### Device details

We fabricated different metasurface devices with array sizes of 300 × 300, 400 × 400, 500 × 500, and 600 × 600 μm^2^, with a corresponding number of participating nanostructures of 600 × 284, 800 × 378, 1000 × 472, and 1200 × 567, respectively. The lattice constants of the rectangular arrays are *P*_*x*_ = 500 nm × *P*_*y*_ = 1060 nm. The dimensions of the rectangular gold nanostructures are *L*_*x*_ = 130 nm × *L*_*y*_ = 200 nm, with a thickness of *t* = 20 nm. The lattice is embedded within a homogeneous background *n* ≈ 1.46.

### Fabrication

The metasurfaces are fabricated using a standard metal lift-off process. We start with a fused silica substrate. We deposit a silica undercladding layer using sputtering. We then define the pattern using electron-beam lithography in a positive tone resist bi-layer with the help of a commercial conductive polymer. The mask was designed using shape-correction proximity error correction^60^ to correct for corner rounding. Following development, a thin adhesion layer of chromium (0.2-nm thick) is deposited using e-beam evaporation, followed by a layer of gold deposited using thermal evaporation. Lift-off is performed, and a final protective silica cladding layer is deposited using sputtering. The initial and final silica layers are sputtered using the same tool under the same conditions to ensure that the environment surrounding the metasurface is completely homogeneous. Before characterization, the surface of the device is then covered in index-matching oil. The backside of the silica substrate is coated with an anti-reflective coating to minimize substrate-related etalon fringes.

### Characterization

See Supplementary Sec. S5: Experimental set-up for a schematic of the experimental set-up.

#### Coherent light measurements

To measure the transmission spectra, we flood-illuminated all of the arrays in the sample using a collimated light beam from a broadband supercontinuum laser source. The wavelength spectrum of the source ranges from *λ* = 470 to 2400 nm. The beam comes from normal incidence along the *z*-direction with light polarized in the *x*-direction. The incident polarization is controlled using a broadband linear polarizing filter. Light transmitted by the metasurface is then imaged by a *f* = 35 mm lens, and a 100-μm pinhole is placed in the image plane to select the desired array. The transmitted light is collected in a large core (400 μm) multimode fiber and analyzed using an optical spectrum analyzer and is normalized to a background trace of the substrate without gold nanostructures. The resolution of the spectrometer is set to 0.01 nm.

#### Incoherent light measurements

Here the experiment goes as above, but the samples are excited using a collimated tungsten-halogen light source (ranging from *λ* = 300 to 2600 nm) and a 400-μm pinhole.

## Supplementary information

Supplementary Information

## Data Availability

The data that support the plots within this paper and other findings of this study are available from the corresponding author upon reasonable request.

## Code availability

The code used to analyze the data and the related simulation files are available from the corresponding author upon reasonable request.
