# Supplementary material for: Ultra-high-Q resonances in plasmonic metasurfaces
Source: Nat Commun. 2021 Feb 12;12:974. doi: 10.1038/s41467-021-21196-2 (PMC7881010; doi:10.1038/s41467-021-21196-2)
Supplement: Supplementary file 1 — Supplementary Information [file 41467_2021_21196_MOESM1_ESM.pdf]

Supplementary Information

Below is the supplementary information for *Ultra-high-Q resonances in plasmonic metasurfaces* by M. Saad Bin-Alam, Orad Reshef, Yaryna Mamchur, M. Zahirul Alam, Graham Carlow, Jeremy Upham, Brian T. Sullivan, Jean-Michel Ménard, Mikko J. Huttunen, Robert W. Boyd, and Ksenia Dolgaleva. In Sec. S1, we present supporting material for Fig. 2d and Fig. 3b. In Sec. S2 we determine the type of SLR by looking directly at the polarizability and the lattice sum. Section S3 shows the dependence of the LSPR and SLR behaviours on the particle geometry, produced using FDTD simulations. It also contains additional measurement results for a different metasurface with the same lattice geometry. In Sec. S4, we present a representative image of a fabricated device. In Sec. S5, we describe our experimental setup.

S1 *Q*-factor extraction

Figure S1 shows Lorentzian fits to a series of LSA calculations with varying λ_{LSPR} (see Methods for values). The *Q*-factors extracted from these fits are used to produce the black curves in Fig. 2d. In Fig. S2, we reproduce the fits to the measurements that produced the values for Fig. 3b.

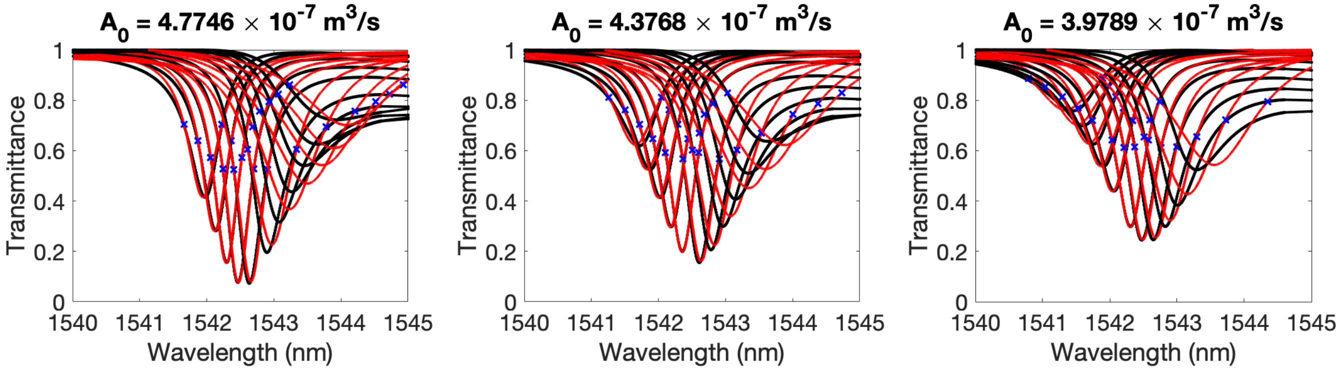

Fig. S1 | Parameter sweeps of LSA calculations. The black curves are calculated using the LSA using the values described in the Methods. The red curves correspond to Lorentzian fits.

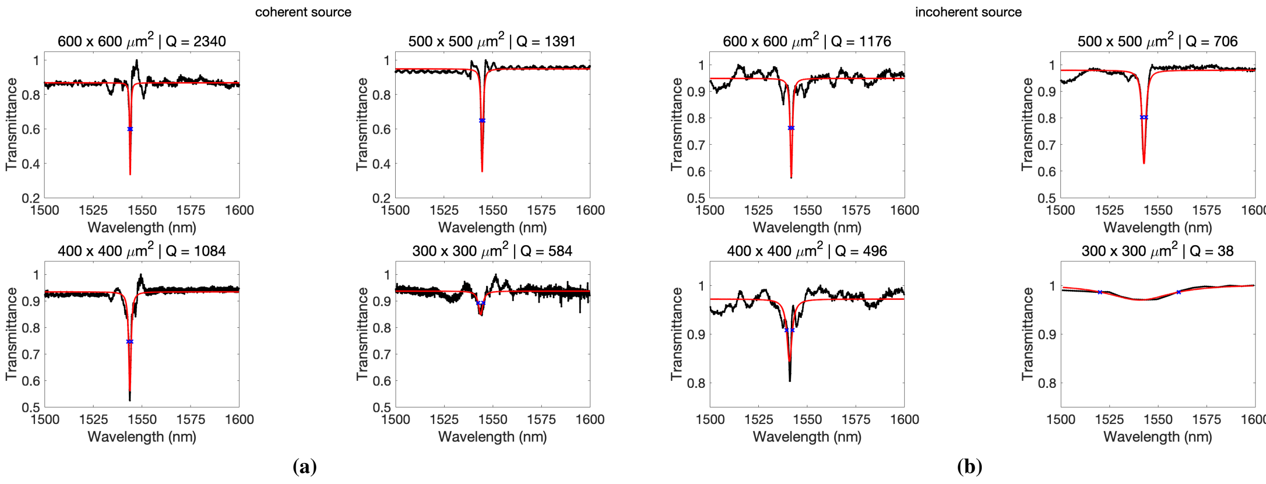

Fig. S2 | Measurements of devices. Measurements (black curves) of the devices described in the main text taken using **a,** a coherent and **b,** an incoherent source. The red lines correspond to Lorentzian fits. The array sizes and extracted *Q*-factors are indicated on the individual figures.

S2 SLR Type

Figure S3 shows the real part of the inverse of the particle polarizability $\mathrm{Re}[1/\alpha]$ as well as the real part of the lattice sum $\mathrm{Re}[\mathbb{S}]$ for the metasurface in Fig. 1. As these two values cross twice near λ_{SLR}, this SLR is considered to be an SLR of the first type according to the nomenclature of Ref.[1].

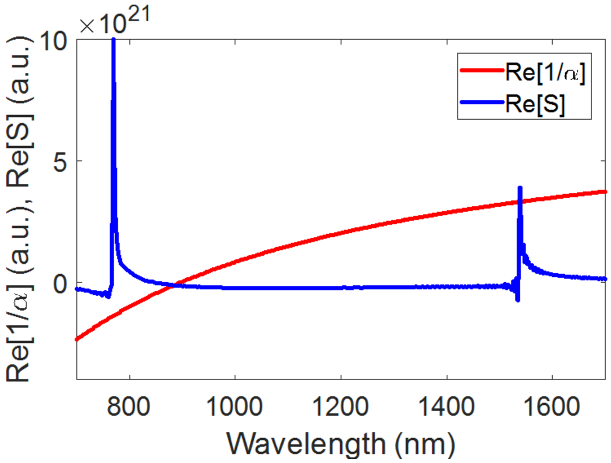

Fig. S3 | SLR type. The real part of the inverse of the particle polarizability $\mathrm{Re}[1/\alpha]$ as well as the real part of the lattice sum $\mathrm{Re}[\mathbb{S}]$ for the metasurface in Fig. 1.

S3 Dependence of SLR behaviour on particle dimensions

To explicitly demonstrate how changing the dimensions of the nanoparticle may affect the properties of the SLR, we perform full-wave simulations in FDTD using a series of particle geometries. Figure S5 depicts the simulation results. Not only the Q-factor, but also λ_{SLR} and the extinction ratio are all affected by changes in the particle dimensions.

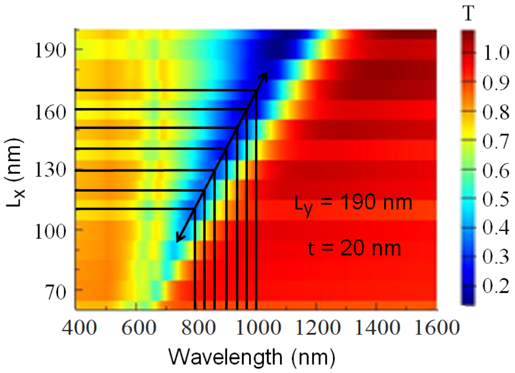

Fig. S4 | Particle dimension vs. LSPR wavelength. λ_{LSPR} (shown in x-axis) linearly increases alongside the particle length L_x towards the light polarization (shown in y-axis). This relation is extracted from full-wave simulations performed with FDTD.

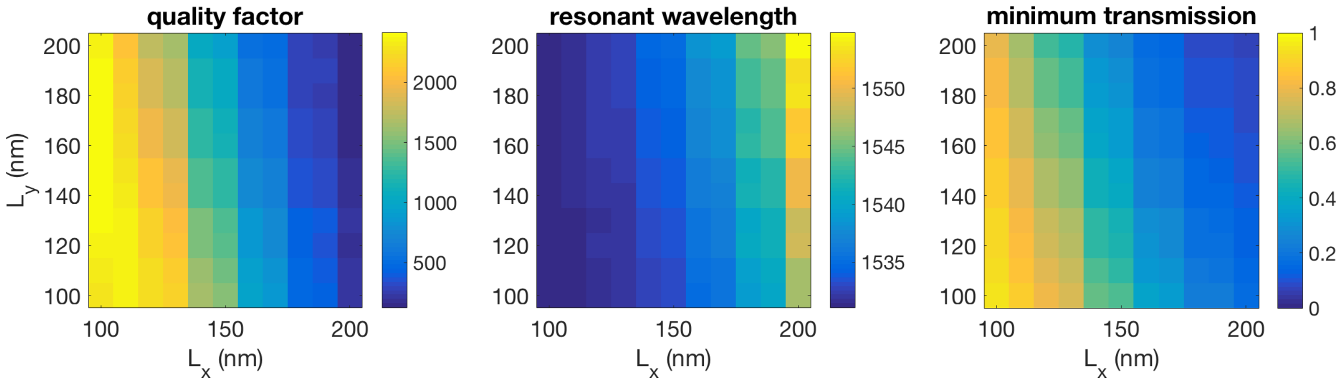

Fig. S5 | Particle dimension sweep. Quality factor Q (left), resonant wavelength λ_{SLR} (center), and minimum transmission as a function of particle dimensions L_x and L_y, extracted from full-wave simulations performed with FDTD.

In Fig. S6a, we present a different $400 \times 400\ \mu\mathrm{m}^2$ array with a nanoparticle geometry of $L_x = 200$ nm, $L_y = 130$ nm. Note that the nanoparticle dimensions are identical to those presented in the arrays above, but due to being rotated by 90 degrees, their resulting particle polarizabilities are completely different. The lattice constants are identical to the arrays presented in the main text, that is, $P_x = 500$ nm and $P_y = 1060$ nm.

The measurements in Fig. S6b were performed using an incoherent source. Here, due to the different polarizability, λ_{LSPR} is red-shifted (1100 nm vs 840 nm), and consequently, the SLR is dramatically affected: in comparison to the matching array in the main text which has an SLR of $Q = 500$, the SLR here only has a $Q = 80$, despite having the same lattice constants and nanoparticle geometries. This further demonstrates the importance of the polarizability to the Q of the SLR.

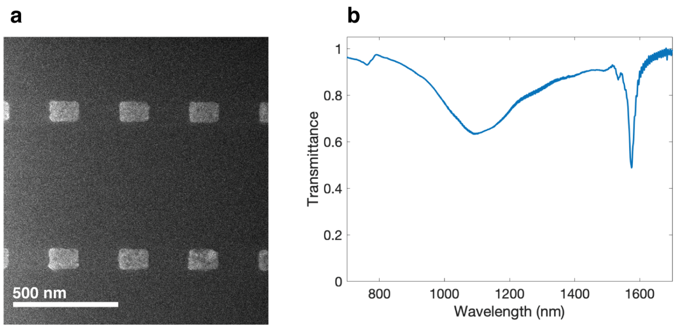

Fig. S6 | Metasurface with same lattice constants but rotated nanoparticles. This device consists of a $400 \times 400\ \mu\mathrm{m}^2$ array with $L_x = 200$ nm, $L_y = 130$ nm, $P_x = 500$ nm and $P_y = 1060$ nm. The SLR is also located at $\lambda_{\mathrm{SLR}} = 1550$ nm, but here $Q = 80$.

S4 Image of the device

Figure S7 shows a typical optical image for one of the devices taken with a bright field microscope. Surrounding the device are large aluminum alignment marks to help locate the device in the experimental setup.

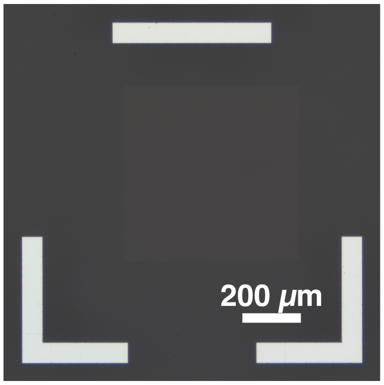

Fig. S7 | Large-area high-Q SLR device. Optical image of a $600 \times 600\ \mu m^2$ array.

S5 Experimental setup

A broadband source is collimated and is polarized using a broadband linear polarizing filter. A first iris is optionally placed to help align the sample in the center of the beam. The beam is then passed through the sample. The surface of the device is imaged using an $f_2 = 35$ mm lens, and a pinhole is placed in the image plane to select the desired array. The transmitted light is collected in a large core (400 μm diameter) multimode fiber and is analyzed using an optical spectrum analyzer.

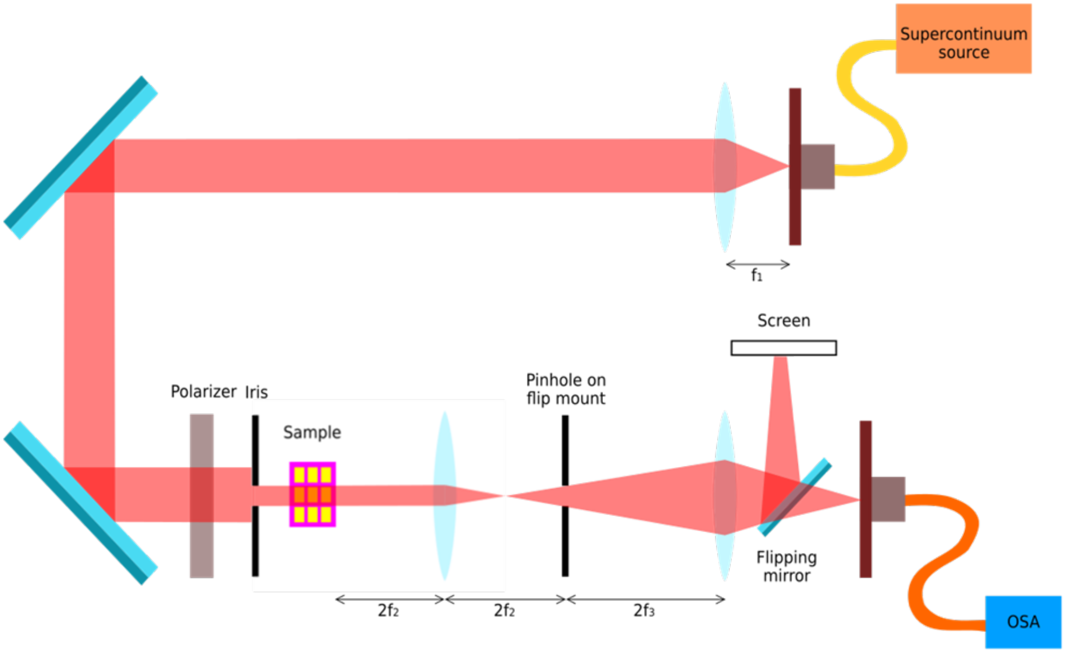

Fig. S8 | Experimental setup. The metasurface is excited by a broadband collimated and polarized beam. Light is collected from the image plane of the metasurface and detected using a camera or a spectrum analyser.

References for Supplementary Information

1. Kravets, V. G., Kabashin, A. V., Barnes, W. L. & Grigorenko, A. N. Plasmonic surface lattice resonances: A review of properties and applications. *Chemical Reviews* **118**, 5912–5951 (2018).
